# Is altering the availability of healthier vs. less-healthy options effective across socioeconomic groups? A mega-analysis

**DOI:** 10.1186/s12966-022-01315-y

**Published:** 2022-07-20

**Authors:** Rachel Pechey, Gareth J. Hollands, James P. Reynolds, Susan A. Jebb, Theresa M. Marteau

**Affiliations:** 1 Nuffield Department of Primary Care Health Sciences, Radcliffe Observatory Quarter, University of Oxford, Oxford, OX2 6GG UK; 2 Department of Public Health and Primary Care, University of Cambridge, Cambridge, CB2 0SR UK; 3 EPPI-Centre, UCL Social Research Institute, University College London, London, WC1H 0NR UK; 4 School of Psychology, Aston University, Birmingham, B4 7ET UK

**Keywords:** Availability, Intervention-generated inequalities, Health inequalities, Socioeconomic position, Food

## Abstract

**Background:**

Availability interventions have been hypothesised to make limited demands on conscious processes and, as a result, to be less likely to generate health inequalities than cognitively-oriented interventions. Here we synthesise existing evidence to examine whether the impact of altering the availability of healthier vs. less-healthy options differs by socioeconomic position.

**Methods:**

Individual-level data (21,360 observations from 7,375 participants) from six studies (conducted online (*n* = 4) and in laboratories (*n* = 2)) were pooled for mega-analysis. Multilevel logistic regressions analysed the impact of altering the availability of healthier options on selection of a healthier (rather than a less-healthy) option by socioeconomic position, assessed by (a) education and (b) income.

**Results:**

Participants had over threefold higher odds of selecting a healthier option when the available range was predominantly healthier compared to selections when the range offered was predominantly less-healthy (odds ratio (OR): 3.8; 95%CIs: 3.5, 4.1). Less educated participants were less likely to select healthier options in each availability condition (ORs: 0.75–0.85; all *p* < 0.005), but there was no evidence of differences in healthier option selection by income. Compared to selections when the range offered was predominantly less-healthy, when predominantly healthier options were available there was a 31% increase in selecting healthier options for the most educated group vs 27% for the least educated. This modest degree of increased responsiveness in the most educated group appeared only to occur when healthier options were predominant. There was no evidence of any differential response to the intervention by income.

**Conclusion:**

Increasing the proportion of healthier options available increases the selection of healthier options across socioeconomic positions. Availability interventions may have a slightly larger beneficial effect on those with the highest levels of education in settings when healthier options predominate.

**Supplementary Information:**

The online version contains supplementary material available at 10.1186/s12966-022-01315-y.

## Background

Diet healthiness is socially patterned such that the most deprived in the population tend to eat less healthy diets with fewer fruit and vegetables [1–4]. This contributes to the substantial socioeconomic inequalities in life expectancy and years lived in good health [5].

Population approaches that tend to rely less on conscious behavioural responses than individual-level interventions have been suggested to be less likely to increase health inequalities [6–8]. These include micro-environmental interventions, which are often characterised as relying largely on non-conscious processes [7, 9]. The extent to which this may hold for particular interventions is unclear.

Availability interventions involve altering the number of instances of a product within the physical micro-environment. These interventions represent a paradigmatic example of micro-environmental interventions that have shown promising evidence of effectiveness [10, 11]. The mechanism by which these interventions operate is not fully known. However, if such interventions work due to increased visual attention and/or salience being given to products with increased availability (hypothesised to operate largely through non-conscious processes), this could lead to equal effectiveness by socioeconomic position (SEP) [12]. Alternatively, if availability acts through individuals’ identifying and selecting their most-preferred option, targeting availability could widen health inequalities, given evidence of pre-existing social patterning in food preferences [13]. The intervention has the potential to further exacerbate the differences between groups given behavioural experiments suggesting that poverty can deplete cognitive resources [14], with cognitive depletion making it harder to resist less-healthy options [15].

To date there is relatively little empirical evidence on the relative effectiveness of availability interventions by SEP, although studies suggest an impact in all SEP groups [16–18]. Some primary research is consistent with responses to availability interventions potentially being stronger for those of higher SEP [17], which could lead to increased inequalities, but other studies find no evidence for a moderating effect of SEP on availability or labelling interventions [19]. This may however reflect a lack of statistical power, given the larger sample size required to detect interaction effects than main effects [20, 21].

Systematic reviews in this area have been limited in their ability to assess the impact of these moderating factors in meta-analyses largely due to the small number of studies available that report such information (e.g. 10). In two recent systematic reviews focused more widely on dietary nudges, one suggested some, but not all, interventions had the potential to increase health inequality [22], while the other found weak evidence that these may be more effective in those with lower SEP [23]. However, conclusions could be influenced by the type of interventions that are predominant in these reviews, such as nutrition labels or logos, given the hypothesis that cognitively-oriented interventions may lead to less equitable outcomes than non-information based interventions. Indeed in both reviews of dietary nudges over half (74 and 56%) were cognitively-oriented interventions. In contrast, a review of the inequalities arising from different types of healthy eating interventions concluded that none of the identified studies targeting environmental changes in specific settings were likely to lead to differential impact by SEP – in contrast to information-based interventions which tended to differentially improve diets of individuals with higher SEP [24].

We have completed a series of studies examining the effect of altering the availability of healthier vs. less-healthy options [16–18, 25]. These have used similar methods allowing the results to be combined in an individual participant mega-analysis. This provides a more powerful test of these potential moderators than allowed by single studies, while access to individual level data allows the use of control variables in a manner not possible with aggregated meta-analyses [26]. A better understanding of whether this non-information based intervention could lead to intervention-generated inequalities may also provide insights relevant to understanding the likely impacts of other micro-environmental interventions.

Accordingly, the current study aimed to evaluate whether the impact of altering the availability of healthier vs. less-healthy options on healthier option selection differed by socioeconomic position. Given different measures that tap into the construct of socioeconomic position may encompass distinct elements underlying the relational nature of socioeconomic position [27], this was investigated separately for different indicators.

## Methods

### Data

Six relevant studies conducted by our research team, four conducted online and two in laboratory settings were included [16–18, 25].

To identify other studies that could contribute data to these analyses, we screened 30 studies identified as potentially eligible as part of the search strategy that was run to update a Cochrane review of availability interventions in June 2021 [10]. No other studies were identified with (i) experimental designs (ii) allowing assessment of availability as a single-component (iii) with selection of a healthier option as a dichotomous outcome variable (to allow mega-analysis), and (iv) that collected data on the SEP of participants.

Characteristics of the six included studies are shown in Table 1. In each case included studies altered the availability of healthier vs. less-healthy foods, and looked at effects on the selection of a healthier option (operationalised as either lower-energy or lower-sugar options). Seven of the nine comparisons showed a significant main effect of availability, although all were in the expected direction. Of the four studies that assessed impact by SEP, the interaction term was statistically significant just for one (with those with lower education being more affected by increased less-healthy options being available).

**Table 1:** Description of studies included in the analysis

**Paper (& study)**	**Setting**	**Sample**		**Intervention**			**Findings** *Odds ratio (OR) (95%CIs)*		**Notes**
		**N (obs)**	**Recruitment criteria**	**Availability type**^a^	**Availability conditions**	**Food target(s)**	**Impact of availability on healthier option selection**	**Differential impact of availability by SEP**	
Pechey & Marteau (2018) [16]	Online; Images of food options	1509 (1509)	UK adults Quotas set by gender, age and occupational status	Absolute & Relative; Range changed	(E) two healthier, two less healthy foods, (MH) six healthier, two less healthy foods, (MLH) two healthier, six less healthy foods	Snacks	*Ref. group E:* MH: 2.0 (1.6, 2.6) MLH: 0.23 (0.17, 0.32)	Main effects (higher education [*ref: 1–4 GCSEs*]): 5 + GCSEs: 1.7 (n.s., 0.8, 3.8); 2 + A levels: 1.4 (n.s, 0.6, 3.5); Degree: 1.8 (n.s., 0.9, 3.6) Interactions (MH [*ref: E*]): 5 + GCSEs: 0.6 (n.s., 0.2, 1.8); 2 + A levels: 1.0 (n.s., 0.3, 3.2); Degree: 0.6 (n.s., 0.3, 1.6) Interactions (MLH [*ref: E*]): 5 + GCSEs: 0.4 (n.s., 0.1, 1.7); 2 + A levels: 0.2 (n.s., 0.0, 1.5); Degree: 0.8 (n.s., 0.3, 2.3)	Other manipulations: cognitive load (high; low)
Pechey, Clarke, Pechey, Ventsel, Hollands, & Marteau (2021) [25]: Study 1	Online; Images of food options	2340 (18,720)	UK adults Quotas set by gender, age and education	Relative; Range kept the same	(E) 50% healthier; (MH) 75% healthier; (MLH) 25% healthier [number of options varied by trial]	Snacks; Drinks	*For fuller shelves condition only*^b^*; Ref. group E:* MH: 1.14 (1.01, 1.28) MLH: 0.95 (n.s., 0.84, 1.07)	Not assessed	Other manipulations: Display layout (emptier^b^; fuller); Manipulation-level (product; category) Participants were explicitly asked about popularity pre-selection
Pechey, Clarke, Pechey, Ventsel, Hollands, & Marteau (2021) [25]: Study 2	Laboratory; Real food options	139 (139)	UK adults Quotas set by gender, age and education	Relative; Range kept the same	(MH) 6/9 healthier & 3/9 less-healthy; (MLH) 3/9 healthier & 6/9 less-healthy	Snacks	*For fuller shelves condition only*^b^*; Ref. group MLH:* MH: 3.3 (n.s., 0.99, 10.9)	Not assessed	Other manipulations Display layout (emptier^b^; fuller)
Pechey, Sexton, Codling & Marteau (2021) [17]	Laboratory; Real food options	417 (417)	UK adults Quotas set by gender, age and education	Absolute & Relative; Range changed	(E) two healthier, two less healthy foods, (MH) six healthier, two less healthy foods, (MLH) two healthier, six less healthy foods	Snacks	*Ref. group E:* MH: 2.5 (1.5, 4.1) MLH: 0.34 (0.20, 0.60)	Main effect (higher education^c^ [*ref: lower*]): 0.54 (n.s., 0.26, 1.12) Interaction (MH [*ref: E*]): 2.50 (n.s., 0.91, 6.83) Interaction (MLH [*ref: E*]): 4.04 (1.31, 12.40)	None
Pechey, Hollands & Marteau (2022) [18]: Study 1	Online; Images of food options	1976 (7904)	UK adults Quotas set by gender, age and education Exclusions: Vegetarians	Relative; Range changed	(MH) 3 healthier; 1 less-healthy; (MLH) 3 less-healthy; 1 healthier	Snacks; Meals	*Ref. group MLH:* MH: 8.9 (7.9, 10.1)	Main effect (higher education^c^ [*ref: lower*]): 1.1 (n.s., 0.9, 1.3) Interaction: 1.0 (n.s., 0.8, 1.2)	None
Pechey, Hollands & Marteau (2022) [18]: Study 2	Online; Images of food options	1078 (2156)	UK adults Quotas set by gender, age and education Exclusions: Vegetarians	Relative; Range changed	(MH) 3 healthier; 1 less-healthy; (MLH) 3 less-healthy; 1 healthier	Meals	*Ref. group MLH:* MH: 9.7 (7.0, 13.5)	Main effect (higher education^c^ [*ref: lower*]): 1.4 (n.s., 0.9, 2.1) Interaction: 1.3; (n.s., 0.8, 2.1)	Greater differences by preference between healthier vs. less-healthy than Study 1

Snacks: Lower-energy (under 100 kcal per pack) vs. higher-energy (over 200 kcal per pack); Drinks: Lower sugar (less than 2.5 g of sugar per 100 ml); higher sugar (2.5 g or more of sugar per 100 ml); Meals: Lower-energy (under 500 kcal per meal); higher-energy (over 500 kcal per meal)

Quotas by age and gender were designed to be representative of the UK population; quotas by SEP recruited equal numbers of higher vs. lower SEP participants

*E* Equal number of healthier vs. less-healthy options, *MH* More healthier than less healthy options, *MLH* More less-healthy than healthy options

^a^Availability types as defined in a conceptual review of availability interventions [12]; (i) Absolute Availability (changing the overall number of options, while keeping the proportions comprised by any subsets of options constant); (ii) Relative Availability (changing the proportion comprised by a subset of options, yet keeping the overall number of options constant); (iii) Absolute and Relative Availability (changing both the overall number of options and the proportions comprised by subsets of options). This was determined for the key comparisons made within the papers, i.e. for Pechey & Marteau (2018) and Pechey, Sexton, Codling & Marteau (2021), this was the comparison between the ‘Equal’ condition and the other availability conditions

^b^“Emptier” trials (layouts designed to reverse the effect of availability by implying previous participants had more frequently selected options that were less available on shelves or trays) were excluded from the mega-analysis; For Pechey, Clarke, Pechey, Ventsel, Hollands, & Marteau (2021) [25]: Study 1: 9306 trials were ‘fuller’ trials; Study 2, 68 participants completed ‘fuller’ trials

^c^In Pechey, Hollands & Marteau (2022) higher education equated to 2 or more A levels; in Pechey, Sexton, Codling & Marteau (2021) [17], higher education equated to degree level or higher

Information on education was collected in all six included studies, and income in all but one study ([25]; Study 2). Other potential indicators of SEP – Index of Multiple Deprivation and occupational status – were each only collected in two of the six studies (Index of Multiple Deprivation [16, 17]; occupational status ([16, 18]; Study 1). Analyses therefore focused on the variables of education and income.

All the studies involved selection rather than purchasing. The number and type of options were matched in one set of studies to those faced by customers in cafeteria settings (based on photos of options offered at a real group of cafeterias), offering a limited number of main meal options [18]. Another involved larger numbers of products, showing drinks and snacks displayed on shelves in a canteen ([25]; Study 1). The remainder – including snack selections made in laboratory studies ([17, 25]; Study 1) – were not designed to mimic a purchasing context.

The raw (individual-level) data were pooled across the six studies, given their similar methodology. Inclusion in the mega-analysis dataset was limited to trials of full shelves or trays (excluding ‘emptier’ trials in Pechey, Clarke et al. [25], which were designed to imply previous customers/participants had selected particular options, so as to reverse the expected pattern for the impact of availability). In total, 21,360 observations from 7,375 participants were analysed (between 1–8 observations per participant). Table 2 shows participant characteristics.

**Table 2:** Participant characteristics

		**Number of participants**	**Mean (s.d.)**
Age		7361	51.0 (16.5)
			**% (of non-missing)**
Gender	Male	3641	49.4
	Female	3723	50.5
	Other	10	0.01
Ethnicity	White	6878	93.9
	Mixed/multiple	105	1.4
	Asian/Asian British	220	3.0
	Black/Black British	88	1.2
	Other	37	0.5
Education^a^	Up to 1–4 GCSEs or equivalent	1616	22.8
	5 + GCSEs or equivalent	1652	23.3
	2 + A-levels or equivalent	1430	20.2
	Degree or higher	2386	33.7
Income^b^	Up to £17,499	1841	26.7
	£17,500-£29,999	1790	26.0
	£30,000-£49,999	1938	28.1
	£50,000 +	1321	19.2

^a^GCSE: General Certificate of Secondary Education, UK qualifications usually sat at around age 16; A-levels: Advanced-level qualifications, usually taken around age 18

^b^Income data were not collected in Pechey, Clarke, Pechey, Ventsel, Hollands, & Marteau (2021) [25]: Study 2

Three Availability conditions were investigated: (1) predominantly healthier, (2) predominantly less-healthy, and (3) equal healthier and less-healthy. Within the predominantly healthier availability condition the available options overwhelmingly comprised 75% healthier options (8794 observations out of 8823; the remaining observations were from trials where the range was 67% healthier). Similarly, nearly all the observations for predominantly less-healthy trials comprised 25% healthier options (8762 out of 8801; with the remaining observations from trials with 33% healthier availability). There were fewer observations (17.5%; *n* = 3736) for ranges with equal numbers of healthier and less healthy options available, which were only included in three studies (see Table 1).

Of the observations, only 485 (2.3%) were laboratory-based (from 2 studies), the remainder from online studies. There were no field trials. The number of products in the range offered varied between 4–64 (mean = 15.6; s.d. 15.6), with 50% of observations offering 4 options. The product range was kept the same following the availability intervention for 43.9% (*n* = 9374) of observations. In terms of food type 60.5% of observations (*n* = 12,928) were for snacks; 28.6% (*n* = 6108) were meals, and 10.9% (*n* = 2324) were drinks.

### Analysis

Multilevel logistic regressions were used to analyse the impact of altering the availability of healthier options on item selection across SEP. Models included three levels, with observations nested within individuals, which were nested within studies – to adjust for the repeated measures designs used in three of studies ([18, 25]: Study 1), and for the potential influence of aspects of individual study design on the behavioural outcome. The primary outcome was a dichotomous variable indicating the selection of a healthier (over a less-healthy) option. The key analyses investigated interactions between the availability variables and socioeconomic position. Inference criteria were set at *p* < 0.01 (Bonferroni’s adjustment).

#### Availability

For the primary analysis, availability conditions (all manipulating Relative Availability) were modelled using dummy variables, with less-healthy as the reference group. Primary analyses only included ‘healthier’ vs. ‘less healthy’ availability conditions, as these appeared in all studies. The ‘equal’ vs. (i) ‘less-healthy’ trials and (ii) ‘healthier’ trials were compared in secondary analyses, with a dummy variable indicating type of availability manipulation (i.e. whether Relative Availability or Absolute&Relative Availability was manipulated).

#### Socioeconomic position

Two analyses were run, looking at different indicators of socioeconomic position (see Table 2) – (1) highest educational qualification and (2) annual household income.

Covariates included in models were: whether the study took place in a laboratory setting (vs. online); whether the product range was kept the same following the availability intervention; the number of products available; the type of food available (modelled using dummy variables); participant age and gender.

#### Alterations to planned analysis

The analysis plan was pre-registered on the Open Science Framework (https://osf.io/gd4y6/). Analyses looking at the impact of interventions by BMI will be reported elsewhere.

Due to issues with model convergence, observations for participants who reported their gender as ‘Other’ (28 observations from 11 participants) were removed from models due to the very small numbers of observations. In addition, food type groupings were re-categorised to be “Meals” vs. “Snacks/Drinks”, to avoid multicollinearity between multiple study-level variables. The covariate ‘hunger’ was included in models in several of the original studies, but was not included in the current primary analyses as this was not collected in one of the studies (sensitivity analyses including this, conducted with the other studies, showed similar results).

For the secondary analyses looking at the ‘Equal’ condition, the variables for whether the product range was kept the same or changed and for whether the study was laboratory vs. online were removed to again avoid multicollinearity (these featured a reduced number of studies for ‘Equal’ trials).

## Results

### Main effects of (a) availability condition and (b) socioeconomic position on selection of a healthier option

#### Availability condition

Compared to selections when the range offered was predominantly less-healthy, participants had over threefold higher odds of selecting a healthier option when the available range was predominantly healthier (odds ratio (OR): 3.82; 95%CIs: 3.54, 4.12).

#### Education

Compared to the most educated participants (degree level education or higher), less educated groups had lower odds of selecting healthier options (up to 4 GCSEs or equivalent: OR: 0.75; 95%CIs: 0.68, 0.84; *p* < 0.001; 5 + GCSEs up to 1 A-level or equivalent: OR: 0.81; 95%CIs: 0.73, 0.90; *p* < 0.001; 2 + A-levels but no degree: OR: 0.85; 95%CIs: 0.76, 0.94; *p* = 0.003).

#### Income

In the model examining main effects only, there was no evidence of differences in the likelihood of selecting healthier options between participants with the highest household incomes (£50,000 per year or higher), compared to those with lower incomes (up to £17,499: OR: 0.91; 95%CIs: 0.81, 1.03; *p* = 0.122; £17,500-£29,999: OR: 0.86; 95%CIs: 0.76, 0.97; *p* = 0.014; £30,000-£49,999: OR: 0.97; 95%CIs: 0.86, 1.09; *p* = 0.615).

### Moderation of the impact of availability condition on selection of a healthier option by socioeconomic position

#### Education

**Fig. 1:**
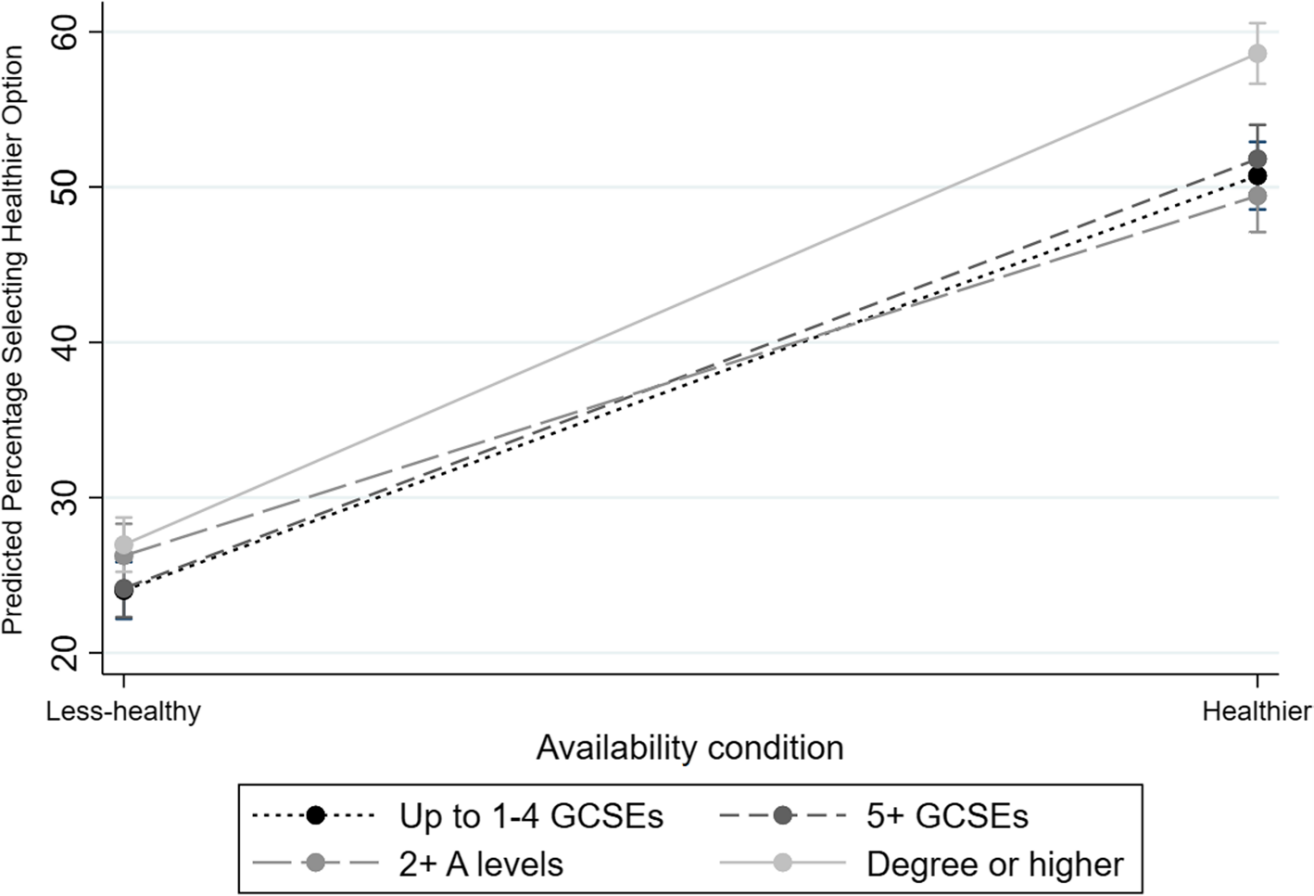
Marginal means (95%CIs) for the proportion predicted to select a healthier option, by availability condition (healthier vs. less-healthy) and highest educational qualification

Figure 1 shows little difference by education when predominantly less-healthy options were available, but a greater proportion of participants with degree-level education may be more likely to select healthier options when predominantly healthier options are available.

When a greater proportion of less-healthy options were available in studies, analyses suggested no evidence of differences in the likelihood of selecting a healthier option at *p* < 0.01 between education levels (Compared to degree level education or higher: up to 4 GCSEs or equivalent (OR: 0.84; 95%CIs: 0.72, 0.97; *p* = 0.018); 5 + GCSEs up to 1 A-level or equivalent (OR: 0.89; 95%CIs: 0.76, 1.03; *p* = 0.112); or 2 + A-levels but no degree or equivalent (OR: 1.05; 95%CIs: 0.90, 1.23; *p* = 0.505)).

The interaction terms suggest that when availability changes to having a greater proportion of healthier options (from a greater proportion of less-healthy options) those with 2 + A-levels but no degree or equivalent are affected less than those with degree level education (OR: 0.67; 95%CIs: 0.56, 0.82; *p* < 0.001). Whilst the other education groups showed a similar direction of effect, these were not significant at *p* < 0.01 (up to 4 GCSEs or equivalent: OR: 0.83; 95%CIs: 0.69, 1.00; *p* = 0.046); 5 + GCSEs up to 1 A-level or equivalent: OR: 0.85; 95%CIs: 0.70, 1.02; *p* = 0.088).

#### Income

**Fig. 2:**
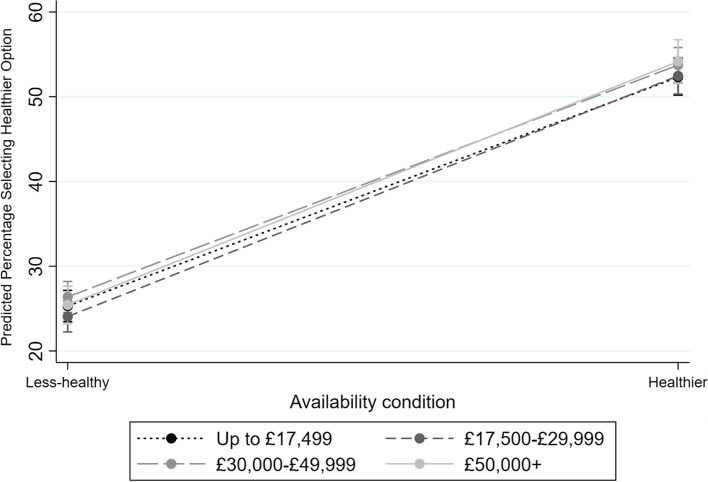
Marginal means (95%CIs) for the proportion predicted to select a healthier option, by availability condition (healthier vs. less-healthy) and annual household income

Interaction analyses suggested no evidence of any interaction effects between income and availability (Fig. 2).

### Secondary analyses

Analyses that also included trials where an equal number of healthier and less-healthy options were offered were conducted, with the ‘equal’ condition as the reference group. These showed a consistent pattern of results to the primary analyses (see Supplementary File 1; Figures S1 and S2, and Supplementary File 2; Models 1b-4b for all model coefficients). Notably, however, these indicate that the difference between the highest educated group and less educated groups was evident only when healthier options were predominant.

## Discussion

The results from this mega-analysis of online and laboratory studies show that over 50% of selections involved a healthier option when the available range was predominantly healthier, compared to around a quarter of selections when the range offered was predominantly less-healthy. Moreover, they suggest that differences related to SEP are limited, with minor differences only observed in relation to education in conditions where healthier options were dominant. For income, there was no evidence of any difference in likelihood of healthier option selection, nor of any differential responding to availability interventions.

This study benefitted from a large sample size due to combining studies, providing more power to test subtle interaction effects that a single study may not be able to identify. Relatedly, another strength was the consistency of both the sets of variables collected and the core elements of study design, allowing a more nuanced investigation of moderating variables – with four levels included for each of the socioeconomic indicators. This consistency is in part due to the studies all being conducted by one research group, however, this could also introduce bias. Replicating these effects using data from other research groups would increase confidence in findings. In addition, limited variation means some elements of study design could not be explored (e.g. different degrees of availability (33% or 67%), or whether relative or absolute availability was altered). Indeed, there were a relatively small number of products available in these studies; if absolute availability has a differential impact by SEP compared to relative availability, then these results may differ in contexts where a greater number of products are available. Future studies exploring the effects of availability interventions and how these vary with the number of products available would be beneficial, particularly as increasing options may increase cognitive load, which has the potential to reduce effects in lower SEP groups.

The key limitation of this mega-analysis is that the included studies comprised online and laboratory studies, with no field studies which might better reflect ‘real-world’ responses. Only two studies included real product selections that participants could immediately consume – both predictably with much smaller sample sizes – so most observations came from online studies with images of products being selected. Social desirability bias could be exacerbated in these contexts, where the consequences of selecting a non-preferred option are minimal. Even in the laboratory studies, these products were offered for free, so may not reflect selections that would be made in a food purchasing context. Moreover, given that diets are made up of a considerable number of such choices, effects are likely to be substantially smaller in experimental studies than studies of dietary patterns. This is possibly reflected in the results for income, where the lack of patterning in healthier food selections may seem surprising, given previous studies have suggested a relationship between income and diet [3, 4], but are consistent with studies of one-off food choices, which have often shown no or little evidence of socioeconomic patterning in selections [16, 25].

The increases in healthier option availability led to increased healthier option selection in all socioeconomic groups, matching the results across each of the online and laboratory studies that contributed data to the mega-analysis. There was, however, some evidence suggesting a minor increase in responsiveness in the most educated, in particular when the majority of options were healthier. This equated to a 31 percentage point increase in selecting healthier options for degree-level participants, compared to a 27 percentage point change for the lowest educated group, i.e. a 4 percentage point difference in the context of a 50 percentage point change in relative availability. This is in line with previous suggestions that predominantly healthier options being available (vs. equal) may lead to more disparity by education (rather than predominantly less-healthy vs. equal), although these analyses lacked power and were not conclusive [17]. Given initial evidence that both preferences and social norms may act as mechanisms underlying the impact of interventions targeting healthier food availability [12, 25], such an effect could be due to those with higher SEP being more likely to prefer healthier options [13], which may also play into, or act alongside, existing social norms within groups. As yet, however, there is relatively limited evidence to support the presence of differences by socioeconomic position for relative preferences for healthier options or social norms with regard to their consumption.

A different pattern of results was found for income. This reflects the results of the review by McGill and colleagues [24], in which the two environment-targeting studies that used income as a measure of SEP found no evidence of differential impact by SEP. (However, studies looking at education or occupation in the McGill review suggested those of lower SEP may see greater impact from interventions, in contrast to the current study). Studies of dietary surveys have found that different measures of socioeconomic position may have independent effects – e.g. showing stronger associations with different food groups or nutritional outcomes – suggesting their additive impacts contribute to lower SEP groups having less-healthy dietary patterns [2–4]. While income could indicate the material resources available to purchase foods – which is less relevant to studies in this mega-analysis where no payments were made – education may be indicative of skills and knowledge to avoid harmful behaviours [3, 4]. Moreover, behavioural experiments suggest that poverty can deplete cognitive resources [14], which may underpin interactions between education and income. Further studies examining moderation by income in contexts where payments are needed would be beneficial. These different facets of SEP can also impact on an individual’s health-related behaviour and subsequent health outcomes in somewhat distinct ways [28]. For example, it has been proposed that lower education may relate more strongly to an individual’s increased likelihood of developing a health issue, while lower income may relate more strongly to subsequent harmful progression of illness [29]. As such, the differential patterning between income and education variables in these analyses could reflect their separate contributions to socioeconomic position.

Further exploration of possible mechanisms that could drive any moderation by socioeconomic position would help to determine how best to utilise availability interventions. If factors such as preferences and social norms play a substantial role, one approach might be to take a stepwise approach to changing availability in contexts where these factors are expected to favour less-healthy options, making smaller changes and allowing time to see if preferences and social norms change in response. Indeed, if changing availability changes social norms, then these interventions may have a wider influence on both diets and minimising inequalities, beyond their direct impact.

The potential for intervention-generated inequalities needs to be considered in the wider context of existing inequalities in food environments, and keeping in mind that this intervention benefitted all SEP groups. Comparing effects by SEP identified in this study assumes that exposure to such scenarios would be equally distributed by SEP, which may not be the case, for example, given those who live in the least affluent areas are most exposed to fast food outlets [30]. Moreover, in retail settings where less-healthy options predominate [31, 32], switching to a more equal distribution of healthier to less-healthy options would not be expected to have any impact on inequalities in food selection by education based on the findings of the current study.

## Conclusion

These analyses suggest that availability interventions can be implemented with minor or no likely adverse impact on health inequalities, particularly when people are selecting food from ranges that are predominantly less-heathy. These interventions show substantial impact on healthier option selection across socioeconomic position, so offer a promising route to increasing diet healthiness across the population.

## Supplementary Information

**Additional file 1: Supplementary file 1:** Figures: Marginal means for the proportion predicted to select a healthier option, by three availability conditions (healthier, equal and less-healthy) and SEP.  

**Additional file 2: Supplementary file 2.** Results of Mega Analysis Models.

## Data Availability

No datasets were generated during the current study. Data analysed is available from the Open Science Framework: Pechey & Marteau (2018): https://osf.io/54rfg/; Pechey, Clarke et al. (2021): https://osf.io/qkh8c/files/; Pechey, Sexton et al. (2021): https://osf.io/x6nhv/; Pechey, Hollands & Marteau (2022): Study 1: https://osf.io/f3thq/; Study 2: https://osf.io/yj89x/.

## Abbreviations

A-level: Advanced-level qualification
GCSE: General Certificate of Secondary Education
OR: Odds ratio
SEP: Socioeconomic position
