# Supplementary material for: Is altering the availability of healthier vs. less-healthy options effective across socioeconomic groups? A mega-analysis
Source: Int J Behav Nutr Phys Act. 2022 Jul 20;19:88. doi: 10.1186/s12966-022-01315-y (PMC9297538; doi:10.1186/s12966-022-01315-y)
Supplement: Supplementary file 1 — Additional file 1: Supplementary file 1: Figures: Marginal means for the proportion predicted to select a healthier option, by three availability conditions (healthier, equal and less-healthy) and SEP. [file 12966_2022_1315_MOESM1_ESM.docx]

**Supplementary File 1: Is Altering the Availability of Healthier vs. Less-Healthy Options Effective Across Socioeconomic Groups? A Mega-Analysis**

*Supplementary Figures*

**Supplementary Figure S1**. Marginal means (95%CIs) for the proportion predicted to select a healthier option, by availability condition (healthier / less-healthy, vs. equal [reference group]) and highest educational qualification

*
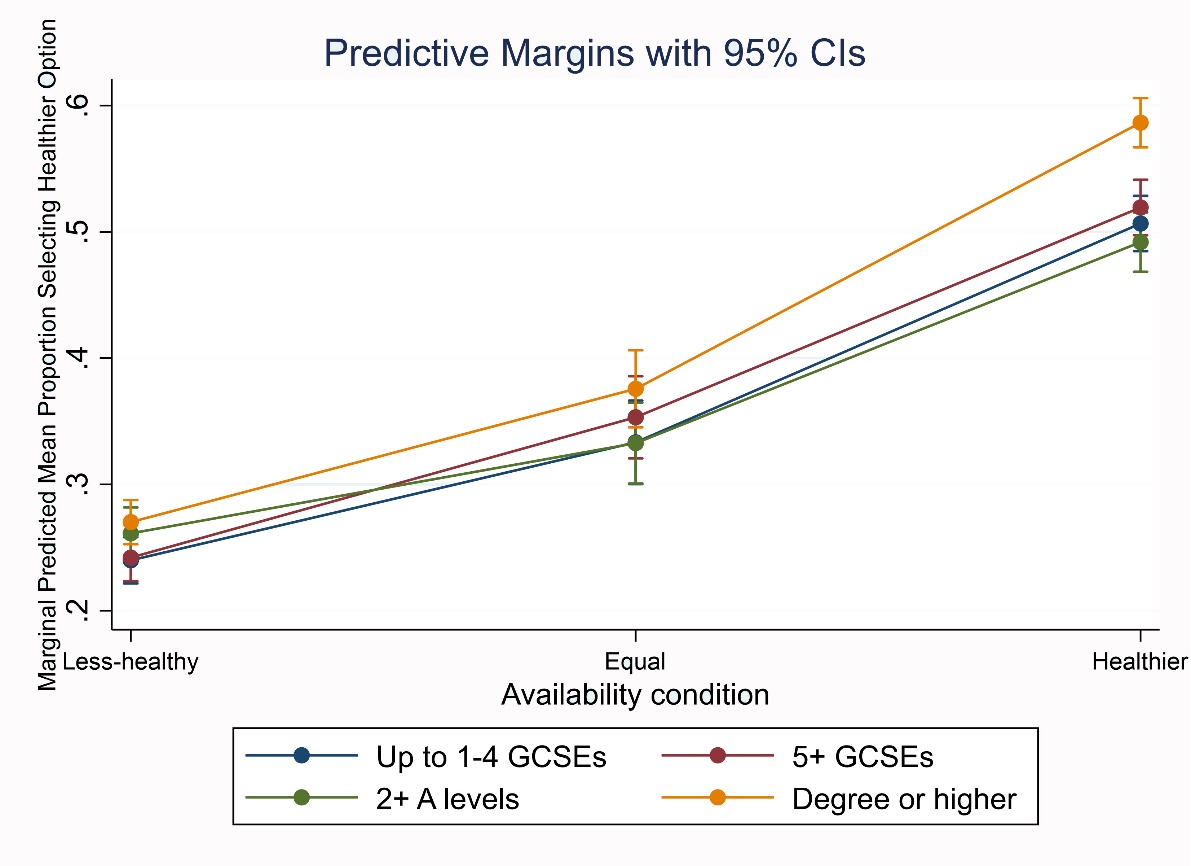
*

No of observations=20,994; participants =7062

**Supplementary Figure S2**. Marginal means (95%CIs) for the proportion predicted to select a healthier option, by availability condition (healthier / less-healthy, vs. equal [reference group]) and annual household income

*
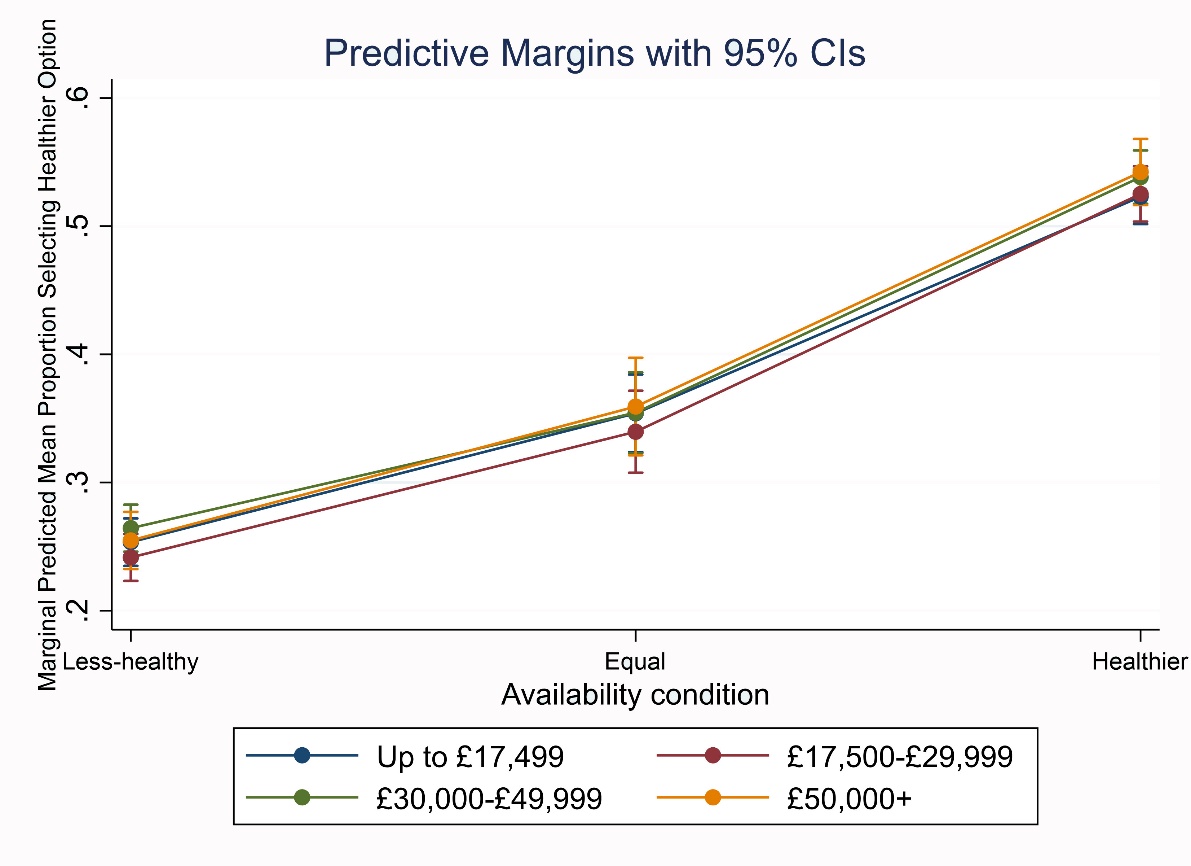
*

No of observations=20,007; participants =6874
